# Does Pretreatment with a Tumor Necrosis Factor Alpha-inhibitor Improve the Outcome After Ischemic Cerebral Infarction? A Case Report

**DOI:** 10.7759/cureus.4089

**Published:** 2019-02-19

**Authors:** Konstantinos Dimitriadis, Michael Wenzel, Grete Buchholz, Andreas Straube

**Affiliations:** 1 Department of Neurology, University Hospital, Ludwig Maximilian University of Munich, Munich, DEU

**Keywords:** stroke, tnfα-inhibitors, blood brain barrier, brain edema

## Abstract

Tumor necrosis factor-α (TNFα) plays a major role in inflammatory and vascular processes after cerebral ischemia. TNFa-Inhibitors have, on the one hand, been associated with thromboembolic events; on the other hand, they may prevent brain edema after stroke or injury. Here, we report on a 38-year old Caucasian male with a history of Crohn´s disease, treated with adalimumab, who presented without brain edema and only minor sequelae after a major ischemic stroke. This case report illustrates two interesting aspects: 1) the treatment with adalimumab could, in that case, be the etiology for the thromboembolic event; and (2) pretreatment with this TNFa-Inhibitor was the most likely reason why the formation of brain edema was suppressed.

## Introduction

Tumor necrosis factor-α (TNFα) is involved in blood-brain barrier (BBB) damage and inflammatory as well as vascular responses after brain ischemia. Mechanisms like the expression of tissue factor, the expression of adhesion molecules for leukocytes, and the activation of matrix metalloproteinases (MMPs) lead to blood-brain barrier disruption and edema [1]. Moreover, the upregulation of TNFα receptor-1 after cerebral ischemia is known to induce apoptosis [2]. TNFα-Inhibitors have rarely been associated with thromboembolic events (TEs) [3-4]. Animal studies suggest that the inhibition of TNFα diminishes brain damage after injury [1,5]. We report on a young patient, with a history of Crohn's disease (CD) treated with adalimumab, who presented without cerebral edema and minor neurological sequelae after a major stroke.

## Case presentation

**Figure 1:**
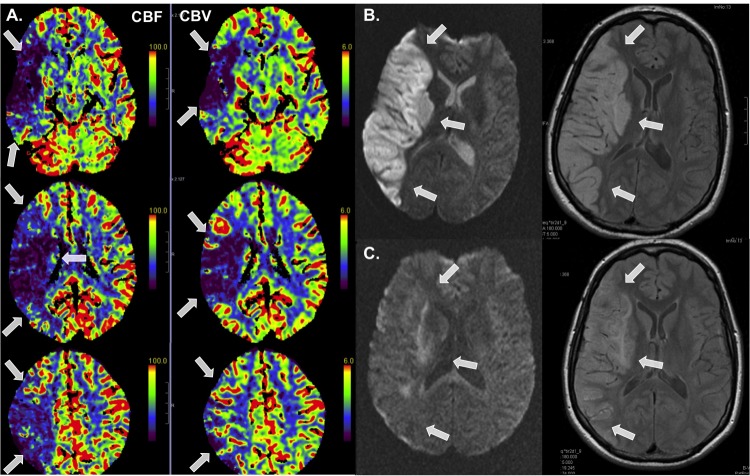
CT and MRI Scans A: Initial computed tomography (CT) perfusion (cerebral blood flow (left) and cerebral blood volume (right)) showing a large perfusion deficit in the right middle cerebral artery (MCA) area with a partial match. B and C: cranial magnetic resonance imaging (MRI, diffusion and flair weighted) two and eight days after the stroke. There is nearly no perilesional edema.

A 38-year old Caucasian male patient with a history of CD (with hemicolectomy four years ago) was admitted to the emergency room after an acute onset of singultus, dysarthria, and severe left-sided sensory motor hemiparesis (NIHSS 15). A head computed tomography (CT) scan revealed an occlusion of the right middle cerebral artery (MCA, M1-section) with a perfusion deficit of the complete territory and a partial mismatch in this area. The occluded MCA was re-opened by combining intravenous thrombolysis (74 mg rtPA) and local mechanical revascularization 165 min after symptom onset. Twelve hours later, the patient surprisingly presented with only a discrete left-sided hemiparesis without hypesthesia and mild dysarthria. Despite the clinical course, a follow-up CT revealed a large infarction comprising two-thirds of the right MCA territory with a slight 3 mm midline shift. Additional magnetic resonance imaging (MRI) scans (Days 2 and 8), however, were not suggestive of significant cerebral edema (Figure 1).

Duplex sonography, 24h-ECG, echocardiography, as well as laboratory and genetic testing for hypercoagulability and vasculitis were unrevealing. There was a history of smoking (18PY). Adalimumab had been started 3Mo prior to admission. The drug concentration in serum on admission was 29.83µg/ml (reference 2.0-33.0µg/ml). Adalimumab therapy was discontinued after the ischemic episode. The patient was discharged with an NIHSS of 1. Three months later the patient showed no motor or sensory deficits and resumed his employment.

## Discussion

The findings of this case can be discussed along two lines.

First, it is somewhat unusual that a healthy young subject without any cardiac precondition develops an MCA occlusion. Therefore, it is necessary to discuss if CD itself or its treatment are of pathophysiological relevance in this context. An association of adalimumab treatment and cerebral ischemia has not been described yet. Nevertheless, TEs have been observed in about 4.5% of patients treated with TNFα-antagonists [3-4,6]. Our patient was on adalimumab only for 3 Mo, without concomitantly receiving corticosteroids and/or methotrexate and/or cyclooxygenase-2 (COX-2)-selective inhibitors, no anti-adalimumab, anti-dsDNA, anti-phospholipid, or anti-β(2)-glycoprotein antibodies could be detected.

Eventually, we considered chronic intestinal inflammation due to CD as a potential risk factor for TE. Our patient, however, had been in excellent condition during the three months prior to the infarction, which is why a direct relation seems unlikely.

Secondly, and more remarkably, was the clinical course of the patient. MCA infarctions as expansive as indicated in the MRI images of our patient are usually followed by severe cerebral edema requiring craniectomy. To this date, cerebral infarction size is the major determinant for the probability of cerebral edema [7]. Could it be that the pretreatment with adalimumab prevented cerebral edema in our patient? A rapid increase of TNFα-levels after ischemia was seen, promoting pro-inflammatory capillary endothelial cell responses [8]. Moreover, cerebral ischemia leads to an upregulation of TNFα receptor-1 with consecutive apoptosis [1]. Since adalimumab blocks the interaction of TNFα with cell surface receptors by binding the TNFα-molecule, a protective effect can be assumed. As early as in 1994, Feuerstein discussed the neuroprotective effect of TNFα-inhibition after brain ischemia [8]. In a rat model, the inhibition of TNFα formation 15 min prior and even 60 min after MCA occlusion significantly reduced the infarction size and the clinical deficits without reporting on cerebral edema [9]. King et al. showed that the TNFα-inhibitor R-7050 significantly reduces BBB disruption, which attenuates edema development after intracerebral hemorrhage and improves neurological outcomes [10]. Hosomi et al. showed that TNFα neutralizing mouse antibodies could, at the same time, reduce the MMPs upregulation after ischemic stroke and reduce cerebral edema [2].

## Conclusions

In summary, we present a young patient with a large MCA ischemia of unknown etiology, with a remarkably good clinical course and nearly no cerebral edema. Pretreatment with the TNFα-inhibitor adalimumab might have led to the inhibition of cerebral edema formation.
